# From signal-based to comprehensive magnetic resonance imaging

**DOI:** 10.1038/s41598-021-96791-w

**Published:** 2021-08-26

**Authors:** Gyula Kotek, Laura Nunez-Gonzalez, Mika W. Vogel, Gabriel P. Krestin, Dirk H. J. Poot, Juan A. Hernandez-Tamames

**Affiliations:** 1 Department of Radiology and Nuclear Medicine, Erasmus MC, Dr. Molewaterplein 40, 3015 GD Rotterdam, The Netherlands; 2 GE Healthcare, Hoevelaken, The Netherlands

**Keywords:** Biomedical engineering, Diagnostic markers, Imaging techniques

## Abstract

We present and evaluate a new insight into magnetic resonance imaging (MRI). It is based on the algebraic description of the magnetization during the transient response—including intrinsic magnetic resonance parameters such as longitudinal and transverse relaxation times (T_1_, T_2_) and proton density (PD) and experimental conditions such as radiofrequency field (B_1_) and constant/homogeneous magnetic field (B_0_) from associated scanners. We exploit the correspondence among three different elements: the signal evolution as a result of a repetitive sequence of blocks of radiofrequency excitation pulses and encoding gradients, the continuous Bloch equations and the mathematical description of a sequence as a linear system. This approach simultaneously provides, in a single measurement, all quantitative parameters of interest as well as associated system imperfections. Finally, we demonstrate the in-vivo applicability of the new concept on a clinical MRI scanner.

## Introduction

MRI is extensively used in medicine and biology. It provides meaningful images of different organs and pathologies based on the magnetic properties of specific nuclei present in tissues.

Quantitative MR measures these properties and currently relies on the single species approximation of the nuclear magnetization evolution as described by Bloch’s equation^1^. However, accurate and precise absolute quantification of the properties requires long scan times and is challenging because of the high sensitivity to MR system imperfections such as magnetic field inhomogeneity and radiofrequency pulse inaccuracies^2^. These factors hamper the reproducibility across MR systems.

It is well known that there is no explicit closed-form solution to the Bloch equations for a general, time-dependent magnetic field. There is a solution though—and it is abundantly exploited in MRI technology—for a very simple case, where the magnetic field is constant as stated by Torrey in 1949^3^. We extend this solution from the continuous Bloch equation to a discrete description of an entire imaging sequence. This approach has been partially explored before by other authors^4–9^. We take this initiative further and establish a novel application and framework in the domain of the impending field of parametric transient MR Imaging^10,11^.

For nearly 50 years, MR imaging mainly used steady state MR pulse sequences^12,13^. Such sequences are designed to allow a model with a single magnetization vector per voxel and avoid the magnitude and phase changes that happen during the transient response—as these could introduce undesirable modulations across the k-space. For this reason Hargreaves et al. proposed to reduce the duration of the transient response in “refocused-SSFP” sequences to reach the steady state faster^5^.

In our opinion, insufficient attention has been focused on exploiting the transient response to simultaneously obtain multiple intrinsic MR relaxation times by taking advantage of more complicated signal evolutions than simple exponential recoveries. A patent was filed in 2001 for simultaneous multiparametric estimation from the transient response^14^ and in 2003, Scheffler pointed out the possibility of extracting relaxation parameters from the transient response but without further development or implementation^8^. Assländer et al.^15^, using a similar formalism that Hargreaves, propose to reduce the population of complex conjugate eigenvalues in order to reduce the sensitivity to system imperfections for obtaining only T_1_ and and T_2_ maps without performance comparison to other reference methods.

Recently, Magnetic Resonance Fingerprinting (MRF) has emerged as a new paradigm for quantitative, multi-parametric MR imaging^11^. It takes advantage of the transient response to generate maps of the intrinsic tissue properties such as T_1_, T_2_ and PD. In the original MRF sequence the acquisition parameters are pseudorandomized, which the authors suggest to be essential for generating unique signal evolutions for each tissue. In MRF the signals are matched to an a priori dictionary to find the relaxation properties. This matching procedure avoids the fitting to an explicit signal model based on repeatedly solving the Bloch equations. It thereby essentially extends the successful compressed sensing principle^16^ to the temporal direction.

Some improved alternatives have recently been published, such as Quantitative Transient Imaging (QTI)^10^ that uses a fixed time of repetition and a linearly increasing variable flip angle (vFA) and Magnetic Resonance Field Mapping^17^ which additionally provides B_1_ and B_0_ maps through including multiple B_0_ and B_1_^.^values in the dictionaries. Sbrizzi et al. recently proposed a brute force approach named MR-STAT providing all the relevant maps (T_1_, T_2_, PD, B_1_ and B_0_) that also avoids dictionary matching^18^.

In this work, we propose a new MR method based on a mathematical closed-form description of the magnetization evolution of a single species along a sequence of repetitive blocks which contain RF pulses and readout gradients. This provides four important advantages. First, it enables a comprehensive simultaneous estimation of all intrinsic parameters and avoids confounding by experimental imperfections such as B_0_ inhomogeneities and B_1_ inaccuracies. Second, the analytical description of the sequence of blocks gives a new insight that facilitates the selection of pulse sequence design (TR, FA and phase) to increase sensitivity for all relevant parameters. Third, no dictionary is required to perform the estimation. Fourth, we also show that, the proposed design, based on the insight given by the algebraic mathematical model, avoids the banding artefact—a long-standing issue in the standard balanced Steady State Free Precession (bSSFP) MRI technique.

We propose that the use of the comprehensive MR method described in this work could contribute to standardized MR through the adoption of multiparametric methods in clinical protocols and to increased reproducibility in follow-up and multi-site or multi-vendor studies.

## Theoretical framework—from linear algebra to dissipative coupled harmonic oscillators

We provide an algebraic description of the signal evolution during the entire MR sequence. Our goal is to establish a theoretical framework for quantitative sequences that utilizes a single transient response and can yield quantitative maps for all relevant parameters, intrinsic (${T}_{1}$, ${T}_{2}$, PD) and experimental (${B}_{0}$, ${B}_{1}$) at once. First, we will consider an imaging voxel as homogeneous and represented by a single magnetization vector, i.e. we use a single species model. In general, the assumption of the intra-voxel homogeneity is not valid. However, as we demonstrate, it can be maintained by a careful choice of the acquisition scheme.

### Discrete algebraic description of an MR imaging sequence

**Figure 1:**
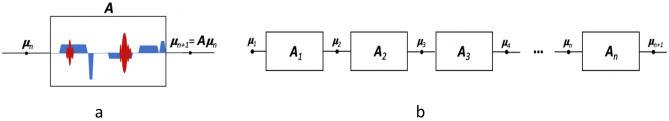
**(a)** Illustration of the “propagator”: a single block of events. μ is the input–output magnetization, in red the RF excitations and in blue the gradients. **(b)** A typical train of blocks in a MR pulse sequence. We will use identical blocks or operators **A** = A_1_ = A_2_ = … = A_n_.

MRI sequences consist of a train of acquisition blocks (Fig. 1). The acquisition blocks alter the magnetization vector. Each block consists of radiofrequency excitations (Fig. 1a, in red), magnetic field gradients (Fig. 1a, in blue) and wait times (Fig. 1a, µ—periods for free precession, absent of RF excitations or magnetic field gradients) each of which can be described as linear operator that acts on the magnetization vector^5^. Hence, the combined effect is also a linear operator called *propagator*.

We focus on a special case when the propagator, ${\varvec{A}}$, is identical along the train of acquisition blocks and has duration ${T}_{R}$. As only the net effect of the propagator is equal, blocks differing in spatial coding k-space trajectories are possible. In this case the magnetization vector ${\varvec{m}}$ evolves according to the recursive equation:

1 $${{\varvec{m}}}_{n+1}={\varvec{A}}{\cdot {\varvec{m}}}_{n}+({\varvec{I}}-{\varvec{A}})\cdot {{\varvec{m}}}_{ss}$$

where ${{\varvec{m}}}_{ss}$ is the steady state magnetization and ${{\varvec{m}}}_{0}$ the initial state. We can focus on the homogeneous recursive Eqs. (5, 9):

2 $${{\varvec{\mu}}}_{n+1}={\varvec{A}}{\cdot{\varvec{\mu}}}_{n},$$

with the solution:

3 $${{\varvec{\mu}}}_{n}={{\varvec{A}}}^{n}\cdot {{\varvec{\mu}}}_{0}$$

where $${{\varvec{\mu}}}_{n}={{\varvec{m}}}_{n}-{{\varvec{m}}}_{ss}$$ .

In comparison, for continuous modeling, the kinetics are described by the differential equation:

4 $$\frac{{\varvec{d}}}{{\varvec{d}}{\varvec{t}}}{\varvec{y}}\left(t\right)={\varvec{B}}\cdot {\varvec{y}}(t),\mathrm{ with the solution}: {\varvec{y}}(t)={{\varvec{A}}}^{t/{T}_{R}}\cdot {\varvec{y}}(0))$$

**Figure 2:**
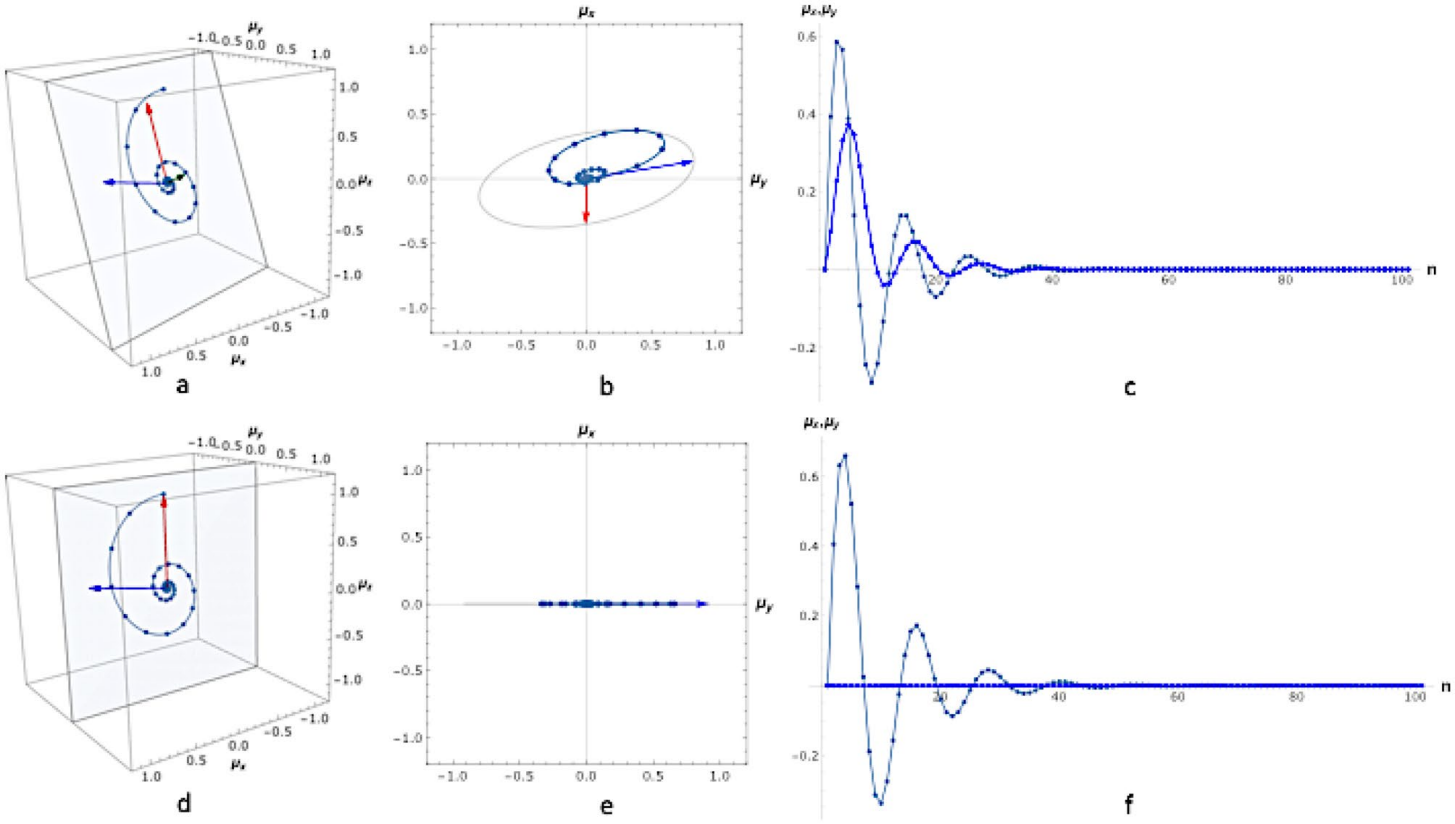
(**a–c)** The repeated block consists of an excitation with flip angle $\mathrm{\alpha }$=30º, $\upbeta$=14º (accumulated phase as a result of off-resonance during ${\mathrm{T}}_{\mathrm{R}}$,${\mathrm{T}}_{\mathrm{R}}$ =10 ms), relaxation times are ${\mathrm{T}}_{1}$= 878 ms, ${\mathrm{T}}_{2}$= 47.5 ms. ${\mathbf{n}}_{1}$ and ${\mathbf{n}}_{2}$ span the plane of oscillation. The magnetization vector always points to a point of this plane. Only the orientation of the plane is fixed throughout the evolution; it shifts parallel to ${\mathbf{n}}_{3}$. Figure **(a)** 3D trajectory, **(b)** x–y projection (transversal plane), **(c)** x and y component (signals measured in quadrature). Figures **(d–f)** similar to **(a–c)** with on-resonance ($\upbeta$=0º). The plane spanned b. ${\mathbf{n}}_{1}$ and ${\mathbf{n}}_{2}$ is the x–z plane throughout the entire evolution. The points represent the magnetization difference vector and its evolution along the sequence. The continuous line is the corresponding continuously parametrized $\mathbf{y}\left(\uptau \right)={\mathbf{A}}^{\uptau /{\mathrm{T}}_{\mathrm{R}}}{{\varvec{\upmu}}}_{0}$ . The quantity $\mathbf{y}\left(\uptau \right)$ satisfies the differential equation: $\frac{\mathrm{d}}{\mathrm{dt}}\mathbf{y}\left(\mathrm{t}\right)=\mathbf{B}\cdot \mathbf{y}\left(\mathrm{t}\right)$ with $\mathrm{exp}\left(\mathbf{B}\right)={\mathbf{A}}^{1/{\mathrm{T}}_{\mathrm{R}}}$. $\mathbf{y}\left(\uptau \right)$ is not the fully continuous trajectory of the magnetization $\upmu$. However, $\mathbf{y}\left(\uptau \right)$ and $\upmu$ are equal at the discrete time points: $\mathbf{y}\left(\uptau =\mathrm{n }{\mathrm{T}}_{\mathrm{R}}\right)={\upmu }_{\mathrm{n}}$ .

The constant matrix coefficients in the differential equation and the recursive equation are related as ${e}^{{\varvec{B}}}={{\varvec{A}}}^{1/{T}_{R}}$^19^. Figure 2 depicts both the continuous and the discrete solutions.

In the case of an imaging sequence consisting of $k$ consecutive excitations and free precession periods, ${\varvec{A}}$ takes the following form:

5 $${\varvec{A}}=\prod_{j=1}^{k}({{\varvec{E}}({\tau }_{j})\cdot {\varvec{R}}}_{z}({\beta }_{j})\cdot {{\varvec{R}}}_{y}({\alpha }_{j})\cdot {{\varvec{R}}}_{z}({\gamma }_{j}))$$

with ${{\varvec{R}}}_{{\varvec{s}}}(\vartheta )$ specifying a rotation of angle $\vartheta$ around axis ${\varvec{s}}$ (${\varvec{x}}$, ${\varvec{y}}$ or ${\varvec{z}}$ in Eq. 5), where the $\alpha ,$ $\beta$ and $\gamma$ are a function of the RF flip angle, phase and local off-resonance frequency, and

6 $${\varvec{E}}({\tau }_{j})=\left(\begin{array}{ccc}{e}^{-{\tau }_{j}/{T}_{2}}& 0& 0\\ 0& {e}^{-{\tau }_{j}/{T}_{2}}& 0\\ 0& 0& {e}^{-{\tau }_{j}/{T}_{1}}\end{array}\right)$$

represents the relaxation in the period ${\uptau }_{\mathrm{j}}$, $\sum_{j}{\tau }_{j}={T}_{R}$.

### A real-valued expression for the signal in an MR sequence

In order to exploit the information content of the signal evolution, it is convenient to have a closed-form expression for ${{\varvec{\mu}}}_{n}$ in Eq. (3). This requires an expression for ${{\varvec{A}}}^{n}$, where ${\varvec{A}}$ is a general, real-valued 3 × 3 matrix.

The usual method to derive an expression for ${{\varvec{A}}}^{n}$ is to diagonalize ${\varvec{A}}$ by its eigenvectors provides a simple expression with the eigenvalues ${\lambda }_{1}$, ${\lambda }_{2}$, ${\lambda }_{3}$ :

7 $${{\varvec{A}}}^{n}={{\varvec{V}}}^{-1}\cdot \left(\begin{array}{ccc}{\lambda }_{1}^{n}& 0& 0\\ 0& {\lambda }_{2}^{n}& 0\\ 0& 0& {\lambda }_{3}^{n}\end{array}\right)\cdot {\varvec{V}}$$

where ${\varvec{V}}=[{{\varvec{v}}}_{1}, {{\varvec{v}}}_{2}, {{\varvec{v}}}_{3}]$ is the matrix formed by the eigenvectors of ${\varvec{A}}$ ^5,8^.

A sufficient and necessary condition of the diagonalization is $\mathrm{det}({\varvec{V}})\ne 0$ , i.e. the eigenvectors of ${\varvec{A}}$ are linearly independent. This condition is met if the eigenvalues are distinct, however this is not a necessary condition (multiplicity of the eigenvalues of ${\varvec{A}}$ alone does not preclude diagonalization).

We follow a different method for derivation of the expression of ${{\varvec{A}}}^{n}$. This method does not rely on the diagonalizability of ${\varvec{A}}$ and it is also idependent of the multiplicity of the eigenvalues. The discrete Putzer’s algorithm^20,21^ relies on a consequence of the Cayley-Hamilton theorem: ${{\varvec{A}}}^{n}$ can be expressed as an ($d$-1) order polynomial of ${\varvec{A}}$ $\in {\mathbb{R}}^{d\times d}$_**,**_ in our case with the identity matrix ${\varvec{I}}$_**,**_ ${\varvec{A}}$ and ${{\varvec{A}}}^{2}$. Applying the Putzer’s algorithm the resulting expression for 3 × 3 matrices with distinct eigenvalues is:

8 $${{\varvec{A}}}^{n}={\lambda }_{1}^{n}{\varvec{I}}+\frac{{\lambda }_{1}^{n}-{\lambda }_{2}^{n}}{{\lambda }_{1}-{\lambda }_{2}}\left({\varvec{A}}-{\lambda }_{1}{\varvec{I}}\right)+\frac{{\lambda }_{1}^{n}\left({\lambda }_{2}-{\lambda }_{3}\right)+{\lambda }_{2}^{n}\left({\lambda }_{3}-{\lambda }_{1}\right)+{\lambda }_{3}^{n}\left({\lambda }_{1}-{\lambda }_{2}\right)}{({\lambda }_{1}-{\lambda }_{2})({\lambda }_{1}-{\lambda }_{3})({\lambda }_{2}-{\lambda }_{3})}\left({\varvec{A}}-{\lambda }_{1}{\varvec{I}}\right)\left({\varvec{A}}-{\lambda }_{2}{\varvec{I}}\right)$$

The expression is also valid in its limit for multiple eigenvalues, may any of the eigenvalues as roots of the characteristic polynomial be identical: ${\lambda }_{1}\to {\lambda }_{2}$, ${\lambda }_{1}\to {\lambda }_{3}$, ${\lambda }_{2}\to {\lambda }_{3}$. ${\varvec{A}}$ is a product of rotation and dilation operations, represented by a non-singular, real valued matrix. The eigenvalues of ${\varvec{A}}$ can always be expressed as ${\lambda }_{1}=\rho {e}^{i\varphi }$, ${\lambda }_{2}=\rho {e}^{-i\varphi }$, and ${\lambda }_{3}=\eta$, where $\rho$ and $\eta$ are real valued, and $\varphi$ is either real or purely imaginary. ${\varvec{A}}$ has one real- valued eigenvalue and its real eigenvector (${\lambda }_{3}$ and ${{\varvec{v}}}_{3}$), but the other two eigenvalue and eigenvector pairs are usually complex conjugates. It is also relevant to realize that ${\rho }^{2}\cdot \eta$ is the determinant of the propagator which depends on the intrinsic T1 and T2 and it is essentially the total dissipation during the entire propagator block. This amount can be voxel-wise obtained and it can be reconstructed as a map. This map is characteristic of the specific harmonic oscillator and we will refer to it as the HO (harmonic oscillator) map, or image, for the rest of the document.

Substituting the eigenvalues in Eq. (8), with a somewhat lengthy, but otherwise straightforward derivation we get:

9 $${{\varvec{A}}}^{n}={\rho }^{n}\cdot \mathrm{sin}\left(n\varphi \right)\cdot {{\varvec{M}}}_{1}{+ \rho }^{n}\cdot \mathrm{cos}\left(n\varphi \right)\cdot {{\varvec{M}}}_{2}+{\eta }^{n}{\cdot {\varvec{M}}}_{3},$$

The real-valued matrices ${{\varvec{M}}}_{j}$ are:

$${{\varvec{M}}}_{1}=\frac{1}{\rho \cdot \mathrm{sin}\varphi }\cdot {\varvec{A}}-\frac{cos\varphi }{\mathrm{sin}\varphi }\cdot {\varvec{I}}+\left(-\frac{\eta }{\rho }+\mathrm{cos}\varphi \right)\cdot {{\varvec{M}}}_{3}$$

$${{\varvec{M}}}_{2}= {\varvec{I}}-{{\varvec{M}}}_{3}$$

10 $${{\varvec{M}}}_{3}=\frac{1}{ {\rho }^{2}+{\eta }^{2}-2\rho \cdot \eta \cdot cos\varphi }\left({{\varvec{A}}}^{2}+{\rho }^{2}{\varvec{I}}-2\rho \cdot cos\varphi \cdot {\varvec{A}}\right)$$

The expression for the special cases when two or all three eigenvalues are identical can be derived in the limit, e.g. $\varphi \to 0$, $\rho {e}^{i\varphi }\to \eta$.

So, the explicit discrete expression of the magnetization evolution as in Eq. (3) is :

11 $${{\varvec{\mu}}}_{n}={\rho }^{n}\cdot \mathrm{sin}\left(n\varphi \right)\cdot {{\varvec{n}}}_{1}{+ \rho }^{n}\cdot \mathrm{cos}\left(n\varphi \right)\cdot {{\varvec{n}}}_{2}+{\eta }^{n}{\cdot {\varvec{n}}}_{3},$$

where the real-valued normal mode^22^ vectors ${{{\varvec{n}}}_{1}={\varvec{M}}}_{1}\cdot {{\varvec{\mu}}}_{0}$, ${{\varvec{n}}}_{2}={{\varvec{M}}}_{2}\cdot {{\varvec{\mu}}}_{0}$ and ${{\varvec{n}}}_{3}={{\varvec{M}}}_{3}\cdot {{\varvec{\mu}}}_{0}$ depend on the initial state ${{\varvec{\mu}}}_{0}$ .

At this point it is important to make two important remarks.

First, in order to clarify the terminology: these normal modes are not the eigenvectors (sometimes also called normal modes, especially if ${\varvec{A}}$ is a symmetric matrix). They are always real valued and represent special directions in real 3D space, along which motion is defined in Eq. (11).

Second, it is important to point out that the matrix expressions in Eq. (10). include a projection matrix: $\mathrm{Re}\left({\varvec{A}}-{\lambda }_{1}{\varvec{I}}\right)=\left({\varvec{A}}-\rho cos\varphi {\varvec{I}}\right)$. Based on this one, we can predict that the normal modes can also have zero length. This happens to be the case when ${{\varvec{\mu}}}_{0}$ is in the null-space of a matrix ${{\varvec{M}}}_{1}$, ${{\varvec{M}}}_{2}$ or ${{\varvec{M}}}_{3}$. The normal modes ${{\varvec{n}}}_{1}$, ${{\varvec{n}}}_{2}$ and ${{\varvec{n}}}_{3}$ form a complete non-orthogonal basis in 3D if ${{\varvec{\mu}}}_{0}$ is not in the nullspace of any of the matrices in Eq. (10).

It is clear from Eq. (11) that ${\varvec{\mu}}$ follows decaying oscillations in two directions ${{\varvec{n}}}_{1}$ and ${{\varvec{n}}}_{2}$ with locked phase and frequency. In the third ${{\varvec{n}}}_{3}$ direction it will follow an exponential decay towards zero.

The detectable signal is a projection of ${{\varvec{m}}}_{n}$ on the plane of detection (${\varvec{x}}{\varvec{y}}$-plane). It is a straightforward calculation to show from Eq. (11), by the usual quadrature detection with a complex expression:

12 $${s}_{n}={a \rho }^{n} {e}^{i(n\varphi +\delta )}+ b {\eta }^{n} {e}^{i \xi }$$

where $a$, $b$, $\delta$ and $\xi$ are real parameters determined by the initial value ${{\varvec{m}}}_{0}$ and the propagator. $\delta$ is the phase difference between transmit and receive. This equation resembles Torreys’ solution of the Bloch Eq. (3) but stretched to the repetitive pulse sequence.

The parameters in Eqs. (11) and (12) are uniquely determined by the ${T}_{1}$, ${T}_{2}$, and PD (intrinsic parameters) as well as the propagator (experimental parameters). Equation (12) highlights the decaying oscillation characteristics of the signal evolution, directly indicating the role of the propagator eigenvalues, parametrized by the intrinsic and experimental parameters.

The evolution of ${\varvec{\mu}}$ (the magnetization difference from the steady state) in three dimensions is illustrated in Fig. 2a,d for a specific propagator and particular experimental conditions: the propagator consists of a single excitation with $\alpha$ flip angle, a phase evolution $\beta$ accumulated during ${T}_{R}$ time during the repetition time (TR) due to off-resonance. The 2D projection over the detection plane is shown Fig. 2b,e, and the detected signal resembling the free induction decay (FID)^3^ is shown in Fig. 2c,f.

Figures a, b and c are in off-resonance condition^4^ and figures d, e and f are in on-resonance condition^5,8^.

## Experimental design

There are three conceptual aspects of our experimental design that render our technique viable in terms of the applicability of a single species model and the sensitivity to intrinsic parameters: (a) Information content of the signal, (b) Single species description, (c) Eigenvalues of the propagator.

### (a) Information content of signal—resonance condition

The description of the motion of ${\varvec{\mu}}$ allows the physical analogy of three independent, linear and damped oscillators, linked to each other only by the initial condition^9,23^. Although ${{\varvec{n}}}_{1}$, ${{\varvec{n}}}_{2}$ and ${{\varvec{n}}}_{3}$ span the space that holds the trajectory of the magnetization, they do not necessarily form a complete basis in 3D. In Fig. 2, the trajectories of ${\varvec{\mu}}$ are depicted in the rotating frame of resonance, where the propagator consists of a single excitation. The case of off-resonance ($\beta \ne 0$) and on-resonance ($\beta =0$) are shown, where $\beta$ is the phase accumulated during a TR. In practice, ${B}_{0}$ can not be fully controlled in an imaging volume, and $\beta$ can take any value in $[\mathrm{0,2}\pi ]$. In the on-resonance case, the last term in Eq. (11) vanishes because ${{\varvec{n}}}_{3}=0$, therefore the signal evolution carries no information about the real eigenvalue $\eta$. The information loss on-resonance can not be avoided with a block containing only one excitation. Hence, a special composite propagator is required.

### (b) Single species model

Equation (11) describes the temporal evolution of the magnetization in a voxel under the assumption that it can be characterized as a single species. There are arguably two reasons why this assumption is not valid: (1) Related to limitations of the imaging technology: Multiple distinct tissue properties in one voxel due to the finite size of voxels or due to experimental imperfections in magnetic and RF fields (${B}_{0}$ and ${B}_{1}$ inhomogeneities). (2) Related to the limitations of the Bloch equations, which are an approximation of the complex microscopic behavior of spin relaxation^1^, excluding multiple-pools for magnetization transfer, diffusion etc. In our experimental design we limit the ${B}_{1}$ imperfections with an appropriate slice profile, and the imperfections in ${B}_{0}$ homogeneity on a more conceptual level in the following section.

### (c) Eigenvalues of the propagator

An important aspect of adopting an analytically described signal evolution is to estimate intrinsic and experimental parameters. As described in Eqs. (11) and (12), the theoretical description relies on the eigenvalues of the propagator.

**Figure 3:**
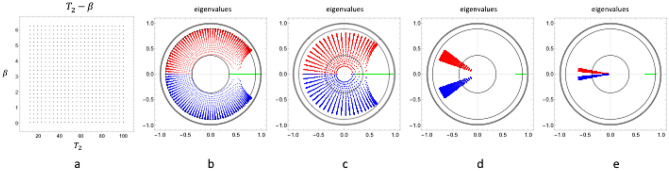
**(a)**${\mathrm{T}}_{2}-\upbeta$ species: parameter space, **(b–e)** are the eigenvalue maps on the complex plane where three eigenvalues belong to each parameter species: red and blue are the complex eigenvalues, green represents the real eigenvalue. Eigenvalues of the repeated ${\mathrm{\alpha }}_{\mathrm{x}}$ propagator are shown in **(b)**. The ${\mathrm{\alpha }}_{\mathrm{x}}-{\mathrm{\alpha }}_{\mathrm{y}}$ propagator with excitation flip angle $\mathrm{\alpha }={30}^{\circ }$ is shown in **(c)**, and in **(d)** “${\mathrm{\alpha }}_{\mathrm{x}}-{\mathrm{\alpha }}_{\mathrm{x}+\updelta }$ ” scheme with $\mathrm{\alpha }={150}^{\circ }$ and $\updelta ={75}^{\circ }$. The eigenvalue space for ${\mathrm{\alpha }}_{\mathrm{x}}-{\upgamma }_{\mathrm{y}}-{\mathrm{\alpha }}_{\mathrm{y}}-{\upgamma }_{\mathrm{x}}$ with $\mathrm{\alpha }={30}^{\circ }$ and $\upgamma ={175}^{\circ }$ is shown in **(e)**. For all maps ${\mathrm{T}}_{1}$=878 ms and ${\mathrm{T}}_{\mathrm{R}}$=12 ms. In **(c)** one eigenvalue point belongs to the two $\upbeta$ and $\upbeta +{180}^{\circ }$ species.

The propagator can also be viewed as a mapping between the parameter space ${(T}_{2},\beta )$ (Fig. 3a) and the space of one of the complex eigenvalues $(\rho ,\varphi )$. As a minimal requirement, this mapping should preserve topology, i.e. it should maintain proximity between points and should be single valued. Otherwise, it would result in indistinguible signal behaviours for distinct ${(T}_{2},\beta )$ species and, consequently, the MR experiment would loose sensitivity to some particular combinations of ${T}_{2}$ and $\beta$.

### Design of a pulse sequence—composite propagator

The propagator that facilitates parametric mapping should fulfill the following requirements:

- The effect of variation of ${B}_{0}$ on the eigenvalues is limited in order to allow the single species description
- The 3D trajectory of ${\varvec{\mu}}$ is maintained at any value of ${\mathrm{B}}_{0}$ ${\mathrm{B}}_{0}$ in order to avoid loss of information (none of the normal mode vectors vanish)
- The mapping between parameter space and eigenvalue space preserves topology in order to distinguish between species by their signal evolution

Figure 3 illustrates the problem of non-preserved topology and also demonstrates proper choices of the scheme, which we use for a set of propagators fulfilling the properties for a better estimation. Also, the inspection of the eigenvalue space provides immediate insight into the signal evolution: the radial distance from the origin determines the decay rate, and the angular position determines the oscillation frequency in the signal along the train of acquisition blocks. Figure 3a shows the grid of ${(T}_{2},\beta )$ pairs that are evaluated with fixed ${T}_{1}$ and excitation flip angle. Figure 3b–e show the resulting eigenvalue space in the complex plane for four different schemes. Figure 3b shows the “${\alpha }_{x}$ ” scheme, in which a block consists of a single excitation with 30° flip angle and readout. Figure 3c shows the “${\alpha }_{x}-{\alpha }_{y}$ ” scheme that consists of two equally spaced excitations along ${\varvec{x}}$ and ${\varvec{y}}$ axis with $\alpha =30$. Figure 3d shows the “${\alpha }_{x}-{\alpha }_{x+\delta }$” scheme consisting of two excitations along the ${\varvec{x}}$ axis and ${\varvec{x}}+{75}^{^\circ }$ axis with flip angle $\alpha =150$. Figure 3e shows the “${\alpha }_{x}-{\gamma }_{y}-{\alpha }_{y}-{\gamma }_{x}$” scheme consisting of four equally spaced excitations with alternating ${\varvec{x}}$ and ${\varvec{y}}$ axis and $\alpha =30$ and $\gamma =175$.

Both the “${\alpha }_{x}-{\alpha }_{y}$ ” scheme (with large flip angle) and the “${\alpha }_{x}-{\gamma }_{y}-{\alpha }_{y}-{\gamma }_{x}$ ” scheme preserve topology and also limit the effect of $\beta$.

Due to the alternating excitation axis, these composite propagators enforce a 3D trajectory of ${\varvec{\mu}}$ (see Eq. 11) for any $\upbeta$. In order to maintain the 3D trajectory for every possible $\beta$ value, a minimum two RF excitations are necessary with different excitation phases. The large excitation angle for the “${\alpha }_{x}-{\alpha }_{y}$” scheme may not be practical due to high demand on RF peak power when a sharp slice profile is used. A split of flip angles into two excitations can be realized in the “${\alpha }_{x}-{\gamma }_{y}-{\alpha }_{y}-{\gamma }_{x}$” scheme: one of sharp slice profile, $\mathrm{\alpha }$, and one with no or very weak slice select gradients, $\upgamma$.

### Numerical optimization—choice of parameters in the selected scheme

The schemes were compared and the parameters of the schemes optimized with a Cramér-Rao lower bound (CRLB) analysis^15^.

Specifically, we evaluated the CRLB for all ten unique $k=4$ schemes differing in the axis around which the RF pulses are played out, using two alternating flip angles $\mathrm{\alpha }\in [20^\circ ,180^\circ ],\upgamma \in \left[10^\circ , 190^\circ \right]$, with steps of $5^\circ$. The simulation used the actual RF profiles as played out on the MR scanner scaled to the nominal flip angle.

For all evaluated settings, the coefficient of variation (CV) of the ${T}_{1}$ and ${T}_{2}$ was evaluated as the division of the square root of the CRLB by the nominal ${T}_{1}$ (800 and 1100 ms) or ${T}_{2}$ (50 and 200 ms) values for $\beta \in [\mathrm{0,2}\pi ]$. These ${T}_{1}$ and ${T}_{2}$ values approximately span most of the commonly quantified tissues^24^.

It was observed that for the schemes with low CV, the CV depended only weakly on ${B}_{0}$. However, for some schemes strong dependence on FA was observed close to the minimum CV. Avoiding those situations, overall the “${\alpha }_{x}-{\gamma }_{y}-{\alpha }_{y}-{\gamma }_{x}$” scheme with $\alpha ={30}^{\mathrm{o}}$ and $\gamma ={175}^{\mathrm{o}}$ was considered to provide the best compromise over the range of ${T}_{1}$, ${T}_{2}$, and ${B}_{0}$.

Hence this scheme is the building block of the new sequence that we named “Multi-Phase balanced, non-Steady State Free Precession” (MP-b-nSSFP).

### Acquisitions

The IRB **(**ethics committee name: “Medische Ethische Toetsings Commissie Erasmus MC”, https://www.erasmusmc.nl/nl-nl/pages/metc) approved the in-vivo study (protocol 2014-096) and the acquisition was carried out after obtaining the informed consent from the volunteers. All experiments were performed in accordance with relevant guidelines and regulations. Only one representative volunteer is presented in this work to show the outcomes obtained. The acquisitions were performed on a 1.5 T clinical scanner (GE Optima MR450w, General Electric Medical Systems, Waukesha, WI) with a 16 channel head and neck coil.

The reference map were obtained with a multiparametric method that currently is a product for different vendors (MAGIC—Magnetic Resonance Image Compilation—for GE scanners). It is based on QRAPMASTER^25^ that uses a multi-echo acquisition of a saturation-recovery sequence combined with a Fast Spin-Echo (FSE) as readout to obtain quantitative maps of T_1_, T_2_, and Proton Density (PD). Once the quantitative maps have been obtained, weighted images are synthesized from these maps. The acquisition was performed in axial orientation (AC-PC) with a TR of 4.7, FOV of 31 cm, and voxel-resolution of 1.2 × 1.2 × 5.0 mm. The total acquisition time of 20 slices covering all the brain was 5 min and 34 s. The phantom was scanned with the same protocol.

Additionally, reference B_1_ and B_0_ maps were obtained for the phantom, in order to compare them from those derived from the method poposed in this work. The B_1_ map is a two-dimensional gradient echo based pulse sequence as described in^26^. B_0_ map is based on 2D GRE pulse sequence repeated two times with different TE as described in^27,28^ .

The images with our sequence—MP-b-nSSFP—were acquired with a Field of View 24 cm, reconstruction matrix 256 × 256, slice thickness 5 mm and 64 rewinded spiral out arms with 512 samples per arm for complete data collection. The images were reconstructed by density compensated non-uniform fast Fourier transform (FFT).

For the proposed MP-b-bSSFP, we acquired 25 repeats of the ${\alpha }_{x}-{\gamma }_{y}-{\alpha }_{y}-{\gamma }_{x}$ scheme (Fig. 3e) α = 30º, γ = 175º, four readouts per block and ${T}_{R}=120$ ms per block with a delay of 3 s between blocks that acquire different spiral arms. The total acquisition time was 6 m and 40 s in this proof of concept study in which no acceleration was used. Due to radiofrequency specific absorption rate (SAR) and time restrictions, the γ pulses were not slice selective and instead surrounded by crushers to suppress out-of-slice signals.

The maps were obtained by for each voxel fitting

$$\theta =\mathrm{arg}\underset{\theta }{\mathrm{min}}{\left|S-Bloch\left(\theta \right)\right|}^{2} \mathrm{with} \theta ={\left[Re\left(S0\right), Im\left(S0\right),E1, E2, \beta , {B}_{1}\right]}^{T}$$

where $S$ is a vector with the complex valued signal of all 100 echoes, $Bloch\left(\theta \right)$ is a single species bloch simulation using hard RF pulses, $E1={e}^{-TR/{T}_{1}}$, $E2={e}^{-TR/{T}_{2}}$ model the longitudinal, respectively transversal magnetization decay, $\beta$ the offresonance induced phase evolution, ${B}_{1}$ a scaling factor for the RF pulses and $S0$ is proportional to proton density and contains the transmit-receive phase.

After fitting, the ${T}_{1}$ and ${T}_{2}$ were computed from $E1$ and $E2$.

The non-linear optimization was started from the best 5 out of 1000 candidate points, pseudo-randomly selected in the range $E1\in [0.9 1]$, $E2\in [0.3 1]$, $\beta \in \left[-\pi \pi \right]$, ${B}_{1}\in [0.5 1.5]$, with linear least squares solution for $S0$. The non-linear optimization was performed with a custom trust region quasi Newton method implemented in MATLAB and the final fit with the lowest cost was returned^29^. The entire fitting procedure took $0.56 s/voxel$ at our workstation (i7-8700 CPU).

An evaluation of the applicability of the model to the acquired data in the presence of intra-voxel B0 dispersion is presented in the supplementary material.

### Demonstration of the signal evolution on a clinical scanner

**Figure 4:**
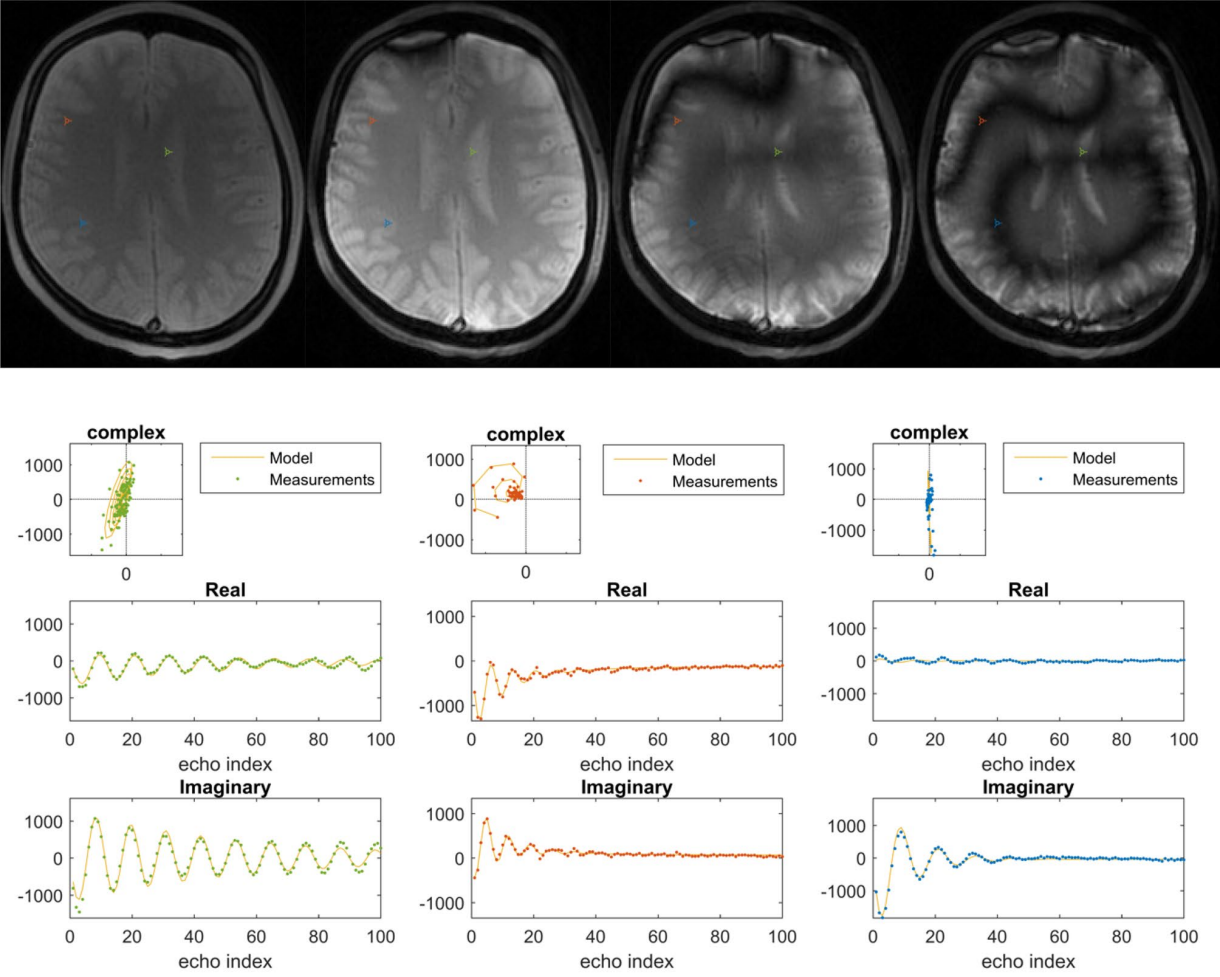
Images in the first row show the transient contrast at time points 1, 3, 5, 7 along the acquisition in a balanced pulse sequence in a “${\mathrm{\alpha }}_{\mathrm{x}}$ scheme”. The second row shows for this sequence the signal as it evolves along the echo train for three voxels (depicted on the anatomical image). The left and right subfigures shows are from the blue and green voxels, where the spins are on-resonance. The middle subfigure (red voxel) shows a spiral in the complex signal plane. This clearly shows the regularity of the evolution. On the right subfigure middle and bottom the orthogonal real and imaginary components are depicted. The frequency of the oscillations is constant, the decay of the amplitudes is exponential. The steady state is not zero. The fitted curves show the fitted harmonic oscillator model.

Figure 4 shows the typical banding artifact present in balanced sequence and how the theoretical model (see Eq. 1) closely matches the data points despite the presence of external field (${B}_{0}$) inhomogeneities.

**Figure 5:**
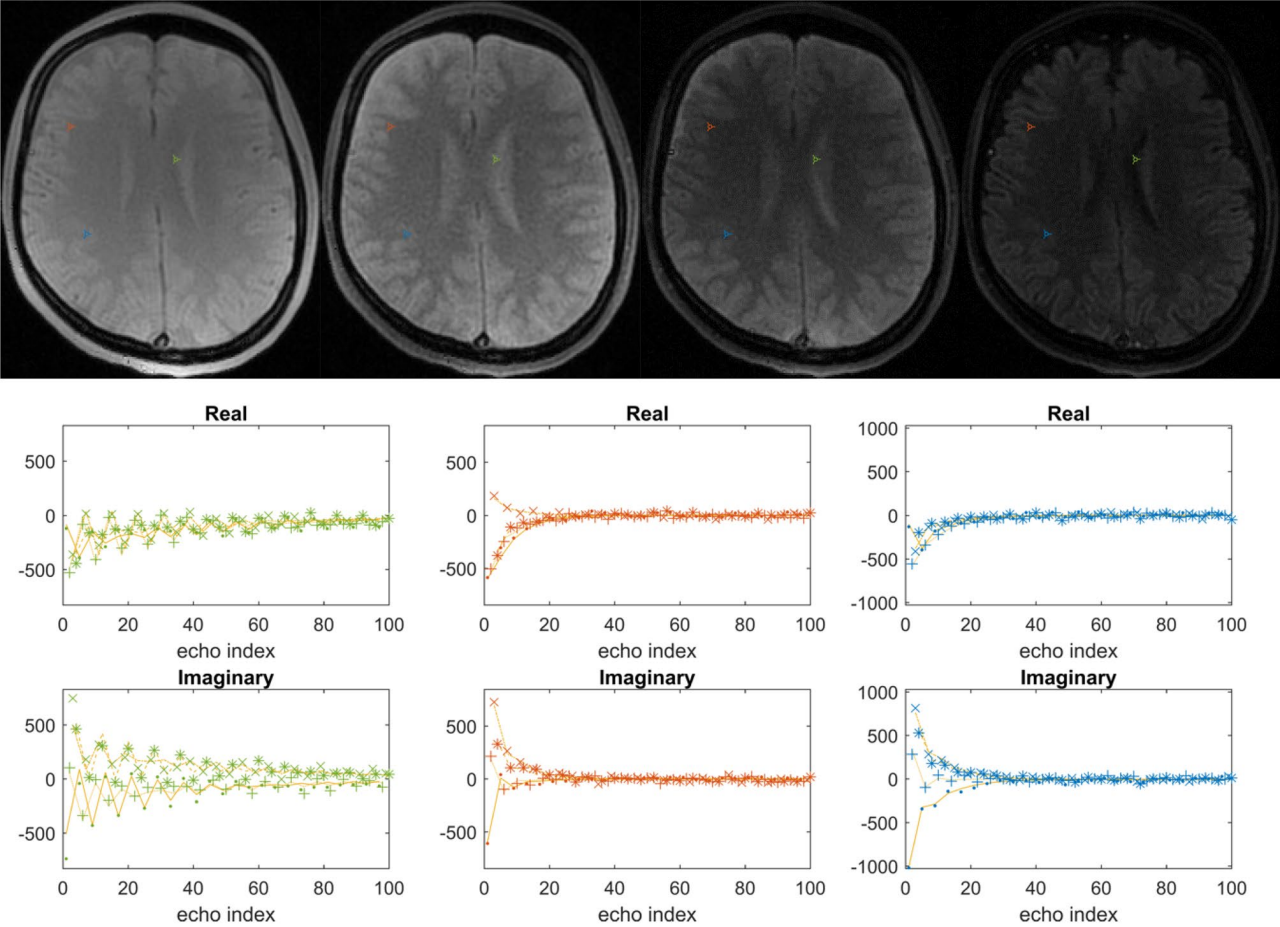
The first row shows the transient contrast at different time points along the acquisition in a balanced pulse sequence for the propagator as a composite of pulses according to the “${\mathrm{\alpha }}_{\mathrm{x}}-{\upgamma }_{\mathrm{y}}-{\mathrm{\alpha }}_{\mathrm{y}}-{\upgamma }_{\mathrm{x}}$ ” MP-b-nSSFP scheme. The second and third rows show the signal and fit for three highlighted voxels from three different tissues. The observations of the four different echoes in each block of the propagator is shown with different symbols (., + , $\times$,*).

Figure 5 shows images and different time points of the signal evolution and the signal evolution for three different tissues using as propagator the “${\alpha }_{x}-{\gamma }_{y}-{\alpha }_{y}-{\gamma }_{x}$ ”scheme in a balanced pulse sequence. The banding artifact is gone and the signal evolution is also described by the same theoretical model (see Eq. 1).

### Standardized phantom and parametric maps

In order to evaluate the accuracy and precision, 12 vials from the Eurospin phantom (https://www.leedstestobjects.com/index.php/phantom/t1-t2-gels/) with known T1 and T2 values were scanned with both methodsMAGIC and MP-b-nSSFP.

**Figure 6:**
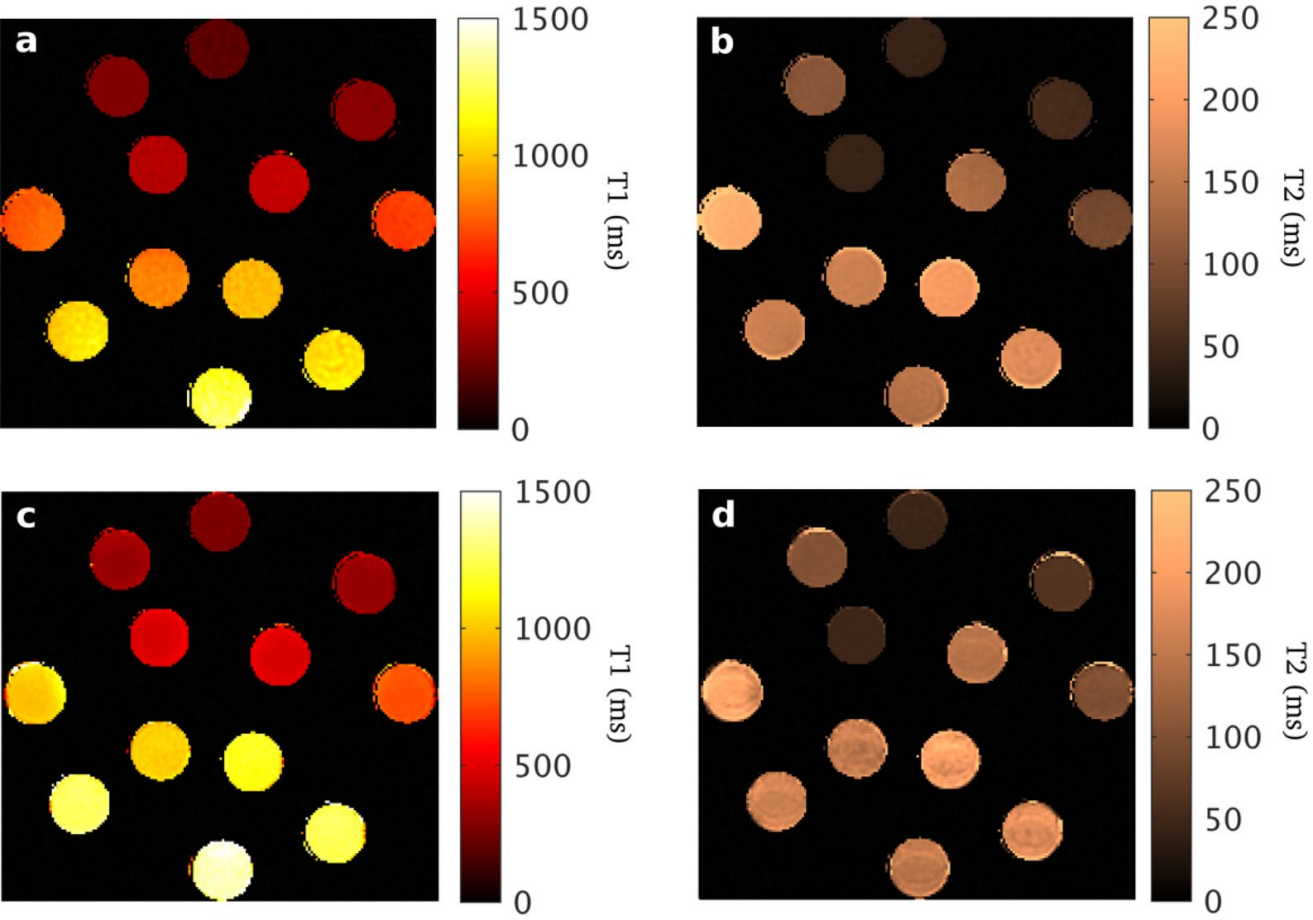
The first row shows the maps from the proposed balanced pulse sequence for the propagator as a composite of pulses according to the “${\mathrm{\alpha }}_{\mathrm{x}}-{\upgamma }_{\mathrm{y}}-{\mathrm{\alpha }}_{\mathrm{y}}-{\upgamma }_{\mathrm{x}}$ ” MP-b-nSSFP scheme ($\mathrm{\alpha }=90;\upgamma =175)$. The second row show maps obtained using conventional MAGIC. **(a,c)** T1 maps. **(b,d)** T2 maps.

Next figure shows the T1 and T2 maps from the MP-b-nSSFP and MAGIC (Fig. 6).

**Figure 7:**
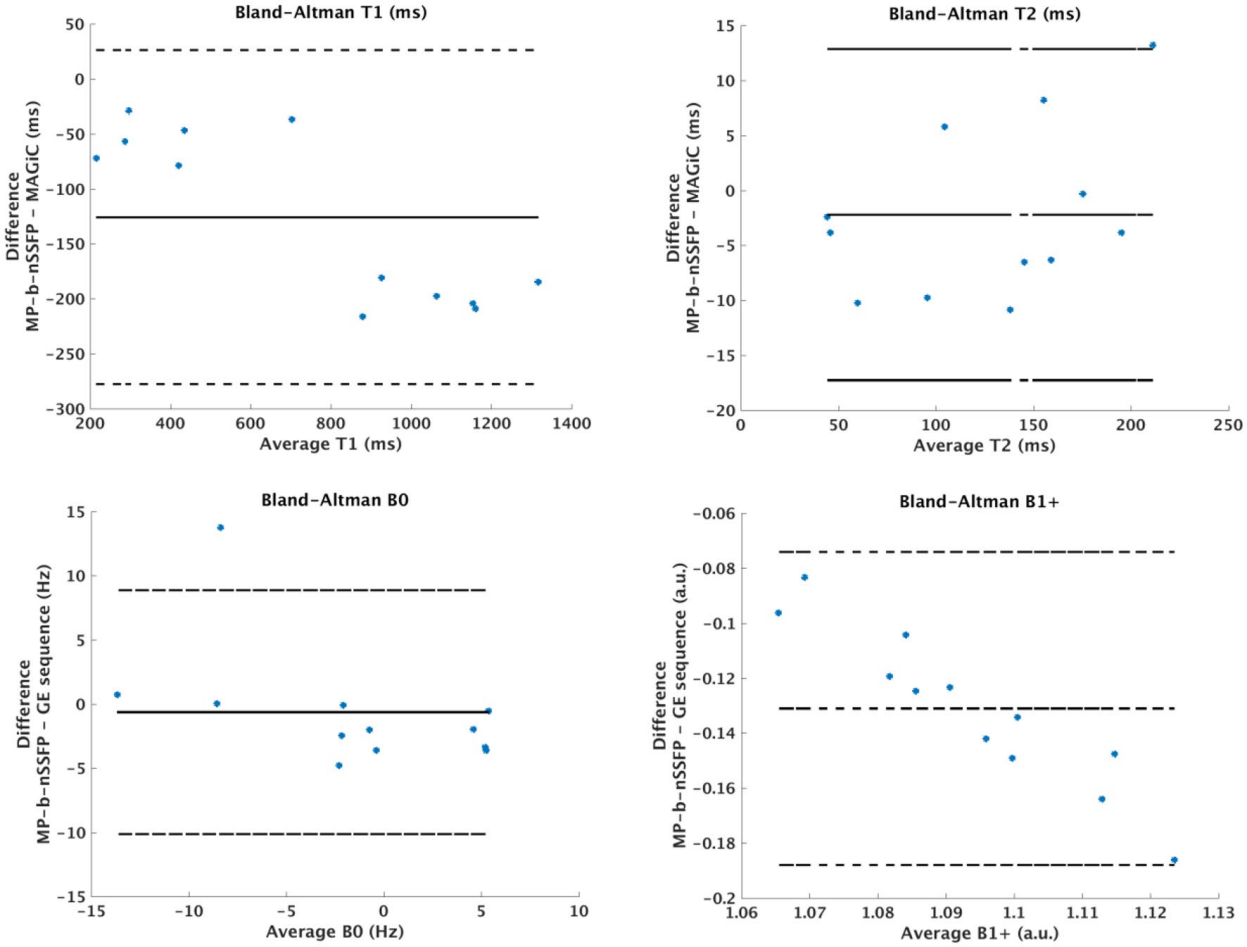
Bland–Altman plots comparing ROI mean ${\mathrm{T}}_{1}$ and ${\mathrm{T}}_{2}$ values of the proposed MP-b-nSSFP sequence with the “${\mathrm{\alpha }}_{\mathrm{x}}-{\upgamma }_{\mathrm{y}}-{\mathrm{\alpha }}_{\mathrm{y}}-{\upgamma }_{\mathrm{x}}$ ” scheme ($\mathrm{\alpha }=90;\upgamma =175)$ and MAGIC.

Figure 7 shows a Bland–Altman comparison between MAGIC and MP-b-nSSFP.

### In-vivo parametric and synthetic maps

**Figure 8:**
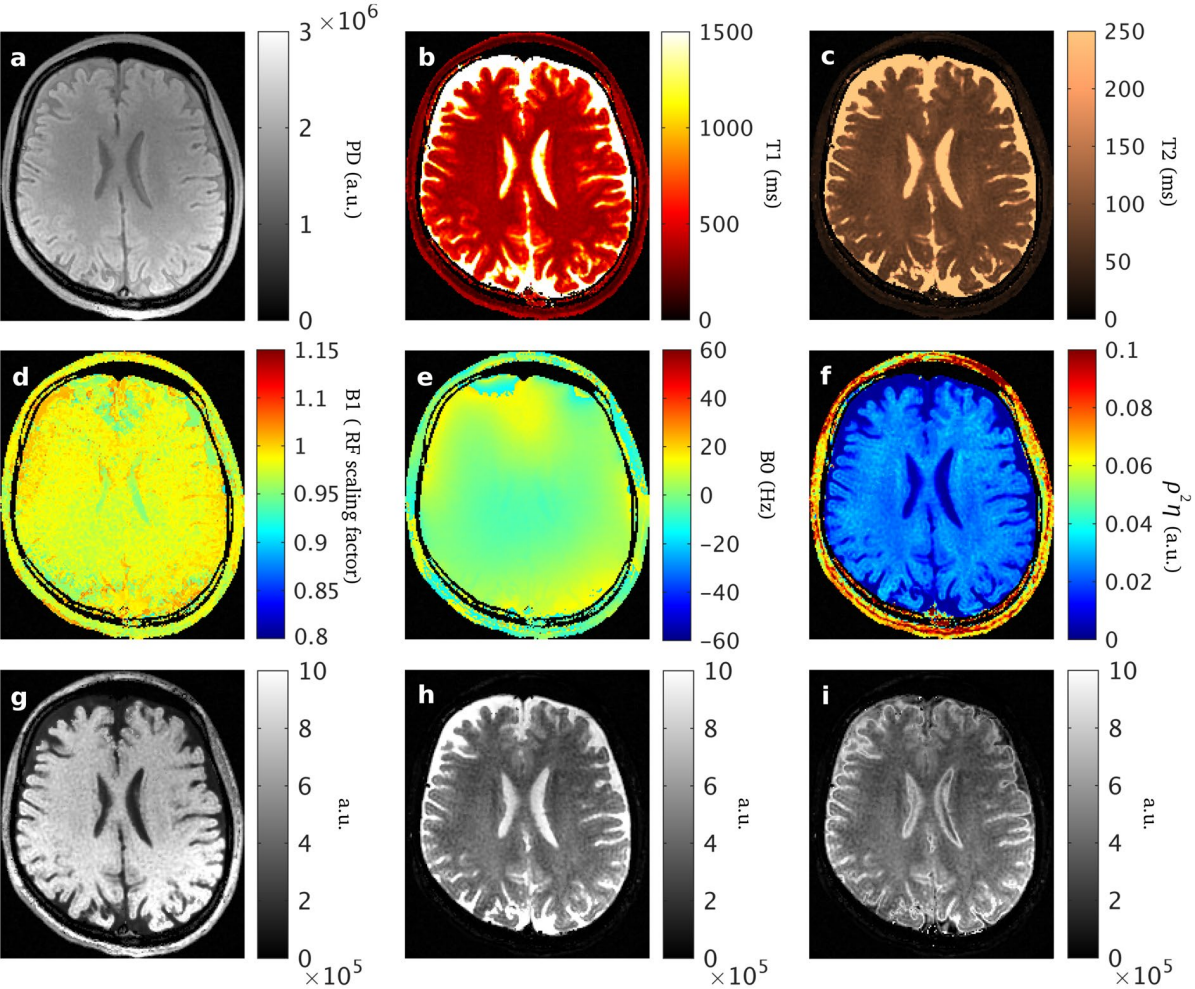
The parametric maps derived with the proposed model are shown estimated from the MP-b-nSSFP sequence with α = 30º, γ = 175º, TR = 30 ms. Top row shows: **(a)** PD (proton density, a.u.); **(b)** ${\mathrm{T}}_{1}$(ms); **(c)** ${\mathrm{T}}_{2}$ (ms); **(d)** ${\mathrm{B}}_{1}^{+}$ (excitation RF field scaling factor); **(e)**${\mathrm{B}}_{0}$ (deviation in static magnetic field; Hz); **(f)** ${\uprho }^{2}\upeta =\mathrm{det}\left(\mathrm{A}\right)={\upvarepsilon }_{2}^{2}{\upvarepsilon }_{1}$ (*HO* harmonic oscillator), **(g)** synthetic T_1_-weighted; **(h)** synthetic T_2_-weighted; **(i)** synthetic T_2_-FLAIR.

Figure 8 shows the results from the MP-b-nSSFP in-vivo scan including the HO maps explained above. Additionally, Fig. 6 shows synthetic weighted images^25^. The T_1_-weighted image was simulated with TE = 20 ms and TR = 300 ms. The T_2_-weighted image was simulated with TE = 120 ms and TR = 4500 ms. The T_2_-FLAIR images were simulated with TE = 120 ms, TR = 15,000 ms and inversion time (TI) equal to 3000 ms. In order to provide a fair comparison we have used with the same protocol described above.

**Figure 9:**
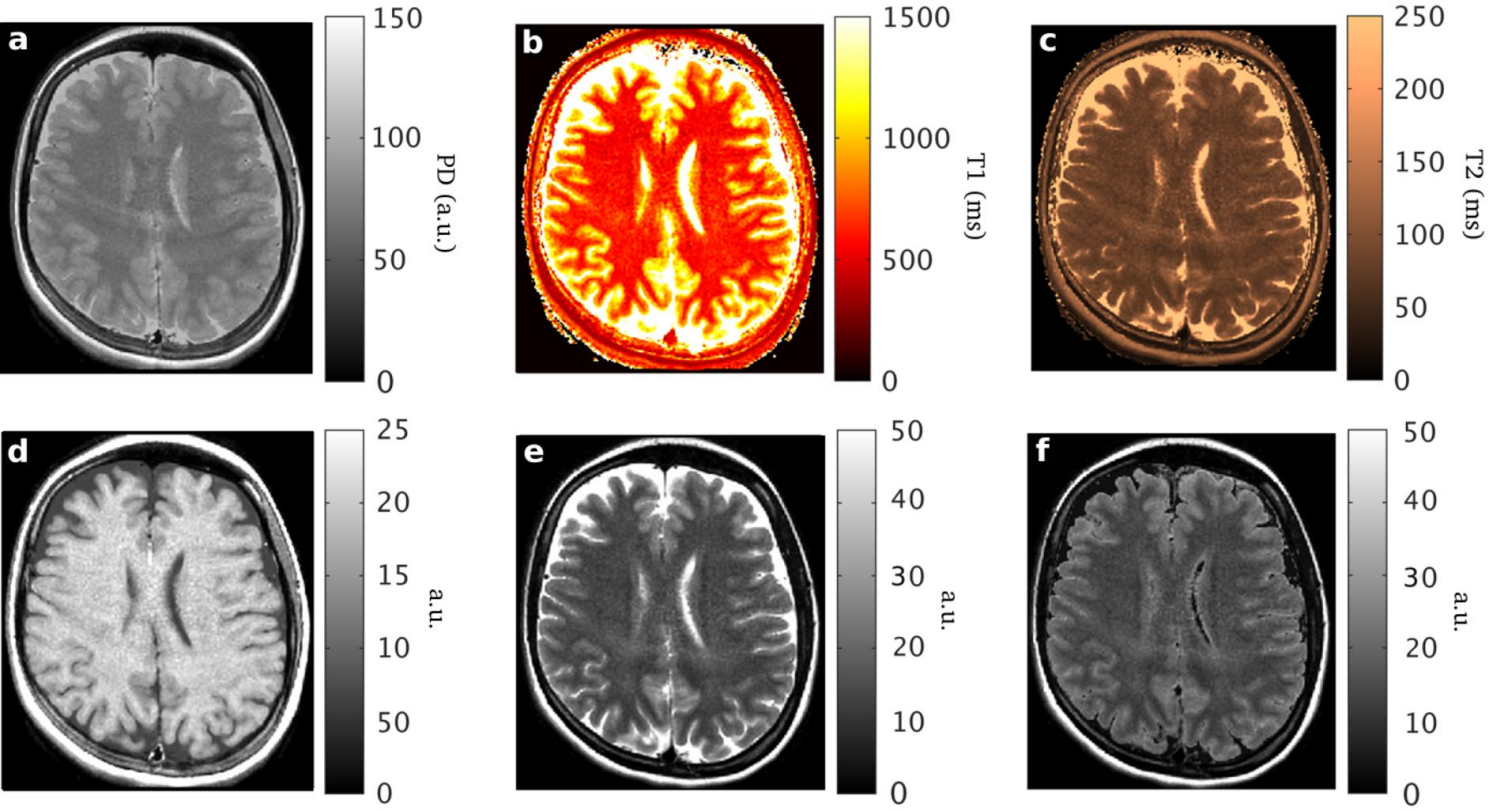
The parametric maps and synthetic images as obtained with MAGIC : **(a)** PD (proton density, a.u.); **(b)** ${\mathrm{T}}_{1}$(ms); **(c)** ${\mathrm{T}}_{2}$ (ms); **(d)** synthetic T_1_-weighted; **(e)** synthetic T_2_-weighted; **(f)** synthetic T_2_-FLAIR.

Figure 9 shows the maps as acquired with MAGIC.

Table 1 summarizes the estimated values of ${\mathrm{T}}_{1}$ and ${\mathrm{T}}_{2}$ in gray and white matter with MP-b-nSSFP and MAGIC and reference values from DESPOT1 and DESPOT2^30^, PLANET^31^ and QRAPMASTER^25^ respectively.

**Table 1:** T1 and T2 values estimated in gray and white matter from the prosposed method, MP-b-nSSFP and MAGIC.

		MP-b-nSSFP	MAGIC	Reference 1	Reference 2	Reference 3
T1	GM	628 (115)	1082 (231)	1065 (51)	813 (54)	1048 (61)
	WM	339 (28)	602 (65)	608 (23)	496 (22)	561 (12)
T2	GM	98 (5)	93 (4)	98 (7)	85(5)	94 (6)
	WM	73 (4)	78 (5)	54 (4)	63(22)	63 (2)

Standard deviations are expressed in parenthesis. Reference columns respectively are: (1) Deoni et al.^30^, (2) Shcherbakova et al.^31^ and (3) Warntjes et al.^25^.

## Discussion

In this work, we conceptually addressed several important aspects for enabling quantitative mapping from the fast transient response in a balanced pulse sequence. With this we extend previous work in this domain^8,14^ to allow composite propagators. It deviates from MR fingerprinting approaches^10,11^ in which experimental parameters are varied along the transient evolution and relaxation parameters are estimated by dictionary matching. Instead, we provide an analytical description of the transient response of a balanced sequence with repetitive blocks. This comprehensively provides simultaneous mapping as well as maps of the experimental conditions.

We proposed a train of acquisition blocks without variations along the train and without suppression of any part of the signal. We showed that a simple and very compact description can be provided for such a sequence. To our knowledge, the resulting real-valued expression has not been previously published.

Additionally, there were two separate aspects that we challenged. One was the impracticality or even impossibility to describe the signal in an analytical form in the transient state, especially when no part of the signal is suppressed (e.g. spoiling). The second aspect was the unfeasibility of using a single species approximation of complex and heterogeneous voxels for the transient evolution of a balanced sequence.

Moreover, we simultaneously addressed a long-standing problem of the balanced Steady State Free Precession technique: banding artefacts. By virtue of the conceptual design of the sequence (i.e.: 3D trajectory maintained by the composite propagator) every point in the image plane bears the same signal evolution characteristics. Along the transient evolution the phase dispersion is limited (see Fig. 3d,e) as the oscillation frequency ($\varphi$, Eqs. 11, 12) is nearly the same for all off-resonance frequencies ($\beta )$. Consequently no (propagating) banding waves in the image space will appear at any time point of the transient response (compare Fig. 4 vs Fig. 5), achieving the desired artefact-free images from balanced MR sequences. This could also be interpreted as a result of the γ pulse which acts as a refocusing pulse, however, the sequence is still sensitive to B_0_ inhomogeneities across the image because the model contains and estimates the intra-voxel phase accrual. To our knowledge, this is a novel achievement that is different from the typical phase cycling in different runs^32,33^.

The resulting signal evolution demonstrates the validity of the single species description despite experimental imperfections.

It can be observed in the results from a standardized phantom (Fig. 7) that most of the results for T_2_ are in good agreement with MAGIC. However, the results for T_1_ are underestimated. Possibly, this is due to slice profile imperfections^34–37^ and finite echo train length as well.

In “In-vivo” experiments, T_2_ results are also in good agreement wih MAGIC. For T_1_, there is a substantial underestimation as well. There are some additional intra-voxel physiological conditions that could be hindering the accuracy in T_1_: intrinsic intra-voxel assymetries^38–41^, water molecules exchanges^42^, diffusion^43^ and magnetization transfer^31,44,45^. If validated and specific enough, such sensitivity to these other phenomena could be exploited for obtaining physiological information such as myelin content. The supplementary material demonstrates that in the current implementation intra-voxel B_0_ dispersion and off-resonance can bias the T_1_ and T_2_ estimation. However, still the effect of B_0_ dispersion is limited as the estimated ${T}_{2}$ value is substantially above the ${T}_{2}^{*}$ value that would be obtained in, for example, a gradient echo experiment. Addressing these T_1_ and T_2_ biases is a topic of further research.

Our method relies on the topological interpretation of an algebraic object (i.e. the propagator: a transformation between the parameter space and the eigenvalue space). With the proposed propagators preserving the topology, the analytical single species description remains valid and allows simultaneous multi-parametric mapping.

It allows the separation of conceptual details of the propagator design and the numerical optimization of the nominal experimental parameters. Regarding the propagator, our method allows single species description even on the presence of B_0_ inhomogeneities based on the range of the appropriate eigenvalues without excluding those complex (unlike Asslander’s proposal^15^). Furthermore, the 3D trajectory of μ is maintained at any value of B_0_ in order to avoid loss of information (none of the normal mode vectors vanish). Moreover, the mapping between parameter space and eigenvalue space preserves topology in order to distinguish between species by their signal evolution. To demonstrate the feasibility on actual scanners, we have selected one of the propagators that fulfills these requirements. In addition to the analytical approach, a numerical optimization of parameters such as flip angles and phases is carried out for the best estimation of T1, T2, PD, B1, B0. So, our novel approach not only enables the estimation of intrinsic parameters, but from the same data and estimation process, one can derive the imperfections of the experimental setup. The macroscopic confounding factors (experimental imperfections such as B_0_ inhomogeneities and B_1_ inaccuracies) are not simply suppressed or excluded in our acquisition, rather they are included in the theoretical description as parameters to be determined (see results “d” and “e” in Fig. 6).

To our knowledge, no other parametric imaging methods provide estimation of this set of intrinsic and experimental parameters simultaneously based on an analytical solution describing the transient response of the magnetization with using dictionaries.The concurrent estimation of intrinsic and experimental imperfections based on the proposed comprehensive analytical model makes this new technique less affected by common system imperfections and could allow for the development of less demanding MR scanners. Based on the robustness of the synchronized estimation of experimental imperfections and parametric maps, synthetic MR images for different clinically relevant contrasts can also be reconstructed without relevant artifacts (see results “g”, “h” and “i” in Fig. 8). As a consequence of these features, our method could contribute to the standardization of MR imaging based on simultaneous multi-parametric mapping and synthetic weighted MR.

## Conclusions

Building on signal-based MR, we provide a complete and comprehensive analytical expression for the signal evolution of a balanced sequence. We extend this solution from the continuous Bloch equation to a discrete description of an entire imaging sequence. The analytical expression relies on a simple, single species model of an imaging voxel which is shown to be appropriate despite the heterogeneity of voxels in-vivo. We demonstrate the importance of an analytical approach in the design of the sequence propagator. This theoretical model could be fitted to experimental data without requiring a dictionary. We simultaneously derive parametric maps of the intrinsic properties (T_1_, T_2_, PD) as well as from imperfections of the experimental parameters (B_0_, B_1_). We demonstrate the feasibility of our method on clinical MRI scanners for in-vivo brain scans.

## Supplementary Information

Supplementary Information.

## References

[1] Bloch F (1946). Nuclear induction. Phys. Rev..

[2] Poorman ME, Martin MN, Ma D, McGivney DF, Gulani V, Griswold MA (2020). Magnetic resonance fingerprinting Part 1: Potential uses, current challenges, and recommendations. J. Magn. Reson. Imaging.

[3] Torrey HC (1949). Transient nutations in nuclear magnetic resonance. Phys. Rev..

[4] Ganter C (2004). Off-resonance effects in the transient response of SSFP sequences. Magn. Reson. Med..

[5] Hargreaves BA, Vasanawala SS, Pauly JM, Nishimura DG (2001). Characterization and reduction of the transient response in steady-state MR imaging. Magn. Reson. Med..

[6] Lukzen NN, Savelov AA (2007). Analytical derivation of multiple spin echo amplitudes with arbitrary refocusing angle. J Magn Reson..

[7] Noh H-R (2020). Analytical solutions and expressions of the propagator for Bloch equations. Int. J. Mod. Phys. B.

[8] Scheffler K (2003). On the transient phase of balanced SSFP sequences. Magn. Reson. Med..

[9] Skinner TE (2018). Comprehensive solutions to the Bloch equations and dynamical models for open two-level systems. Phys. Rev. A..

[10] Gómez PA, Molina-Romero M, Buonincontri G, Menzel MI, Menze BH (2019). Designing contrasts for rapid, simultaneous parameter quantification and flow visualization with quantitative transient-state imaging. Sci. Rep..

[11] Ma D, Gulani V, Seiberlich N, Liu K, Sunshine JL, Duerk JL (2013). Magnetic resonance fingerprinting. Nature.

[12] Carr H (1958). Steady-state free precession in nuclear magnetic resonance. Phys. Rev..

[13] Hahn EL (1950). Spin echoes. Phys. Rev..

[14] Glover, P.M., Hill, R.J. *Apparatus for and Method of Determining Values of Relaxation Parameters*. (Google Patents, 2001).

[15] Assländer J, Novikov DS, Lattanzi R, Sodickson DK, Cloos MA (2019). Hybrid-state free precession in nuclear magnetic resonance. Commun. Phys..

[16] Lustig M, Donoho DL, Santos JM, Pauly JM (2008). Compressed sensing MRI. IEEE Signal Process. Mag..

[17] Korzdorfer G, Jiang Y, Speier P, Pang JN, Ma D, Pfeuffer J (2019). Magnetic resonance field fingerprinting. Magn. Reson. Med..

[18] Sbrizzi A, Heide OV, Cloos M, Toorn AV, Hoogduin H, Luijten PR (2018). Fast quantitative MRI as a nonlinear tomography problem. Magn. Reson. Imaging.

[19] Abrahamson DL (1989). Pursuing analogies between differential equations and difference equations. Am. Math. Mon..

[20] Elaydi SN (2020). An Introduction To Difference Equation.

[21] Putzer EJ (1966). Avoiding the Jordan canonical form in the discussion of linear systems with constant coefficients. Am. Math. Mon..

[22] Goldstein H, Poole C, Safko J (2002). Classical Mechanics.

[23] Skinner TE (2018). Comprehensive solutions to the Bloch equations and dynamical models for open two-level systems. Phys. Rev. A..

[24] Stanisz GJ, Odrobina EE, Pun J, Escaravage M, Graham SJ, Bronskill MJ (2005). T-1, T-2 relaxation and magnetization transfer in tissue at 3T. Magn. Reson. Med..

[25] Warntjes JBM, Leinhard OD, West J, Lundberg P (2008). Rapid magnetic resonance quantification on the brain: Optimization for clinical usage. Magn. Reson. Med..

[26] Sacolick LI, Wiesinger F, Hancu I, Vogel MW (2010). B1 mapping by Bloch-Siegert shift. Magn. Reson. Med..

[27] Prammer MG, Haselgrove JC, Shinnar M, Leigh JS (1988). A new approach to automatic shimming. J. Magnet. Resonan..

[28] Schneider E, Glover G (1991). Rapid in vivo proton shimming. Magn. Reson. Med..

[29] Poot DH, Klein S (2015). Detecting statistically significant differences in quantitative MRI experiments, applied to diffusion tensor imaging. IEEE Trans. Med. Imaging..

[30] Deoni SCL, Peters TM, Rutt BK (2005). High-resolution T1 and T2 mapping of the brain in a clinically acceptable time with DESPOT1 and DESPOT2. Magn. Reson. Med..

[31] Shcherbakova Y, van den Berg CAT, Moonen CTW, Bartels LW (2018). PLANET: An ellipse fitting approach for simultaneous T1 and T2 mapping using phase-cycled balanced steady-state free precession. Magn. Reson. Med..

[32] Shcherbakova Y, van den Berg CAT, Moonen CTW, Bartels LW (2019). On the accuracy and precision of PLANET for multiparametric MRI using phase-cycled bSSFP imaging. Magn. Reson. Med..

[33] Björk M, Ingle RR, Gudmundson E, Stoica P, Nishimura DG, Barral JK (2014). Parameter estimation approach to banding artifact reduction in balanced steady-state free precession. Magn. Reson. Med..

[34] Dieringer MA, Deimling M, Santoro D, Wuerfel J, Madai VI, Sobesky J (2014). Rapid parametric mapping of the longitudinal relaxation time T 1 using two-dimensional variable flip angle magnetic resonance imaging at 1.5 Tesla, 3 Tesla, and 7 Tesla. PLoS ONE.

[35] Ehses P, Seiberlich N, Ma D, Breuer FA, Jakob PM, Griswold MA (2013). IR TrueFISP with a golden-ratio-based radial readout: fast quantification of T1, T2, and proton density. Magn. Reson. Med..

[36] McRobbie DW, Lerski RA, Straughan K (1987). Slice profile effects and their calibration and correction in quantitative NMR imaging. Phys. Med. Biol..

[37] Santini F, Kawel-Boehm N, Greiser A, Bremerich J, Bieri O (2015). Simultaneous T1 and T2 quantification of the myocardium using cardiac balanced-SSFP inversion recovery with interleaved sampling acquisition (CABIRIA). Magn. Reson. Med..

[38] Miller KL (2010). Asymmetries of the balanced SSFP profile. Part I: theory and observation. Magnet. Resonan. Med..

[39] Miller KL, Jezzard P (2008). Modeling SSFP functional MRI contrast in the brain. Magn. Reson. Med..

[40] Miller, K.L. & Jezzard, P. Balanced SSFP profile asymmetries detect small frequency shifts in white matter. *Proceedings of 17th Meeting of the ISMRM, Hawaii*, Vol. 258 (2009).

[41] Miller KL, Smith SM, Jezzard P (2010). Asymmetries of the balanced SSFP profile. Part II: white matter. Magnet. Resonan. Med..

[42] Dharmakumar R, Hong J, Brittain JH, Plewes DB, Wright GA (2005). Oxygen-sensitive contrast in blood for steady-state free precession imaging. Magnet. Resonan. Med..

[43] Bieri O, Scheffler K (2007). Effect of diffusion in inhomogeneous magnetic fields on balanced steady-state free precession. NMR Biomed..

[44] Bieri O, Scheffler K (2006). On the origin of apparent low tissue signals in balanced SSFP. Magnet. Resonan. Med..

[45] Stanisz GJ, Odrobina EE, Pun J, Escaravage M, Graham SJ, Bronskill MJ (2005). T1, T2 relaxation and magnetization transfer in tissue at 3T. Magnet. Resonan. Med..

