# Supplementary material for: From signal-based to comprehensive magnetic resonance imaging
Source: Sci Rep. 2021 Aug 26;11:17216. doi: 10.1038/s41598-021-96791-w (PMC8390767; doi:10.1038/s41598-021-96791-w)
Supplement: Supplementary file 1 — Supplementary Information. [file 41598_2021_96791_MOESM1_ESM.docx]

Supplementary material for:

From Signal-Based to Comprehensive Magnetic Resonance Imaging

Authors: Gyula Kotek*^1^, Laura Nunez-Gonzalez^1^, Mika. W. Vogel^2^, Gabriel. P. Krestin^1^, Dirk H.J. Poot^1^, Juan A. Hernandez-Tamames*^1^.

# ^1^ Department of Radiology and Nuclear Medicine, Erasmus MC, Dr. Molewaterplein 40, 3015 GD Rotterdam, The Netherlands

# ^2^ GE Healthcare, Hoevelaken, The Netherlands

# * These authors contributed equally to this work

## Evaluation of slice profile and intra-voxel B0 dispersion

To evaluate if the proposed model applies to the actual pulse sequence with imperfections in the slice profile and intra-voxel B_0_ dispersion the following simulation study is performed.

Signals of a voxel are generated by an extensive Bloch simulation that includes the full slice/slab selective RF pulse shapes as well as slice selection gradients as they are played out on the scanner for the proposed 25 repeats of the $\alpha_{x}-\gamma_{y}-\alpha_{y}-\gamma_{x}$ scheme with α=30º and γ=175º (it corresponds to the “figure3,e” in the manuscript). Figure S1 shows 1 repeat of the pulse sequence for this scheme.The $\alpha$ pulses have a sharp slice profile and the hard $\gamma$ pulses are crushed.


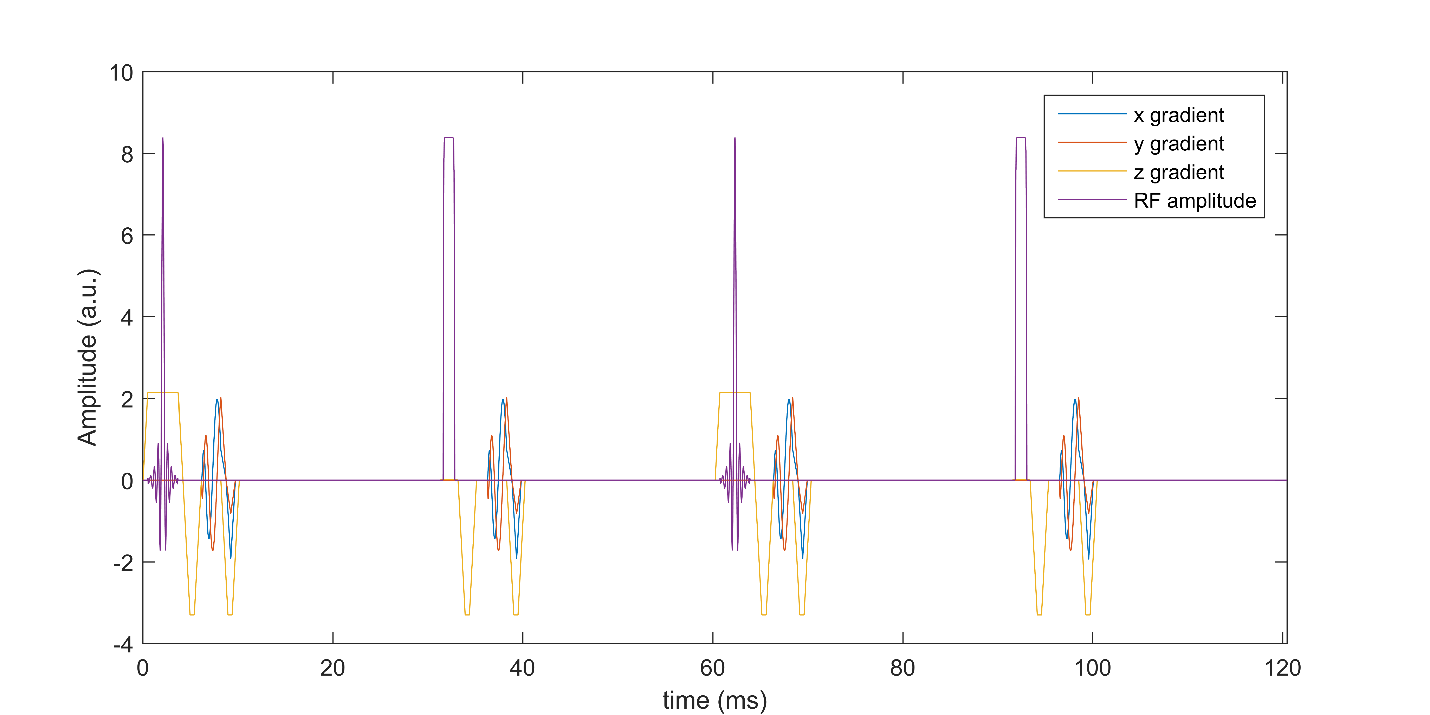


*Figure S1. A single-block of the scheme* $\alpha_{x}-\gamma_{y}-\alpha_{y}-\gamma_{x}$*, with α=30º and γ=175º as played out on the scanner.*

In the simulation, the balanced readout was not included as the simulated spins comprised a single voxel, and the signal was captured at the start of the center-out spiral readout. To simulate the slice profile 400 spins were distributed uniformly over an extend of 1.7 times the slice width of 5mm and the simulation was performed for 1001 values of B_0_ linearly spaced in [-500, 500] Hz. Other simulation parameters:

$PD=1$, $T_{1}=800ms$, $T_{2}=80ms$, and nominal scaling of the RF power.

As realistic model of intra-voxel B_0_ dispersion we constructed Cauchy weighted combinations of the simulated signals with the width corresponding to $T_{2}^{'}=[20, 40, 80, 160, 320, \infty]ms$ with center frequency from -100Hz to 100Hz.

The thus generated signals include the slice profile as generated by the scanner as well as intra-voxel B_0_ dispersion. Subsequently these signals were fitted by the method described in the main manuscript.

## Results

Figure S2 shows the fitted parameters as function of B_0_.

The results with long $T_{2}^{'}$ indicate a low bias in the estimates obtained with the single species model. A relevant effect in the simulation of the current sequence as implemented on the scanner is the different gradient strength during the α and γ pulses. With off-resonance, the gamma pulse shifts slice position much more than the alpha pulse. Hence effectively for |B_0_|>25Hz the gamma pulse is lower inside the slice selected by the α pulse. This seems to primarily cause a bias towards lower values of $T_{1}$. With 3D acquisitions or a different design of the RF pulses this could be remedied.

The results shows that intra-voxel dispersion is causing bias to the estimated parameters. For low |B_0_| the $T_{1}$ seems to be positively biased when $T_{2}^{'}<2 TR$. $T_{2}$ is underestimated when substantial intra-voxel B_0_ dispersion is present (i.e. low $T_{2}^{'}$). However, as aimed for in the design, the dispersion is limited and hence the estimated $T_{2}$ value is substantially above the $T_{2}^{*}$ value that would be obtained in, for example, a gradient echo experiment.


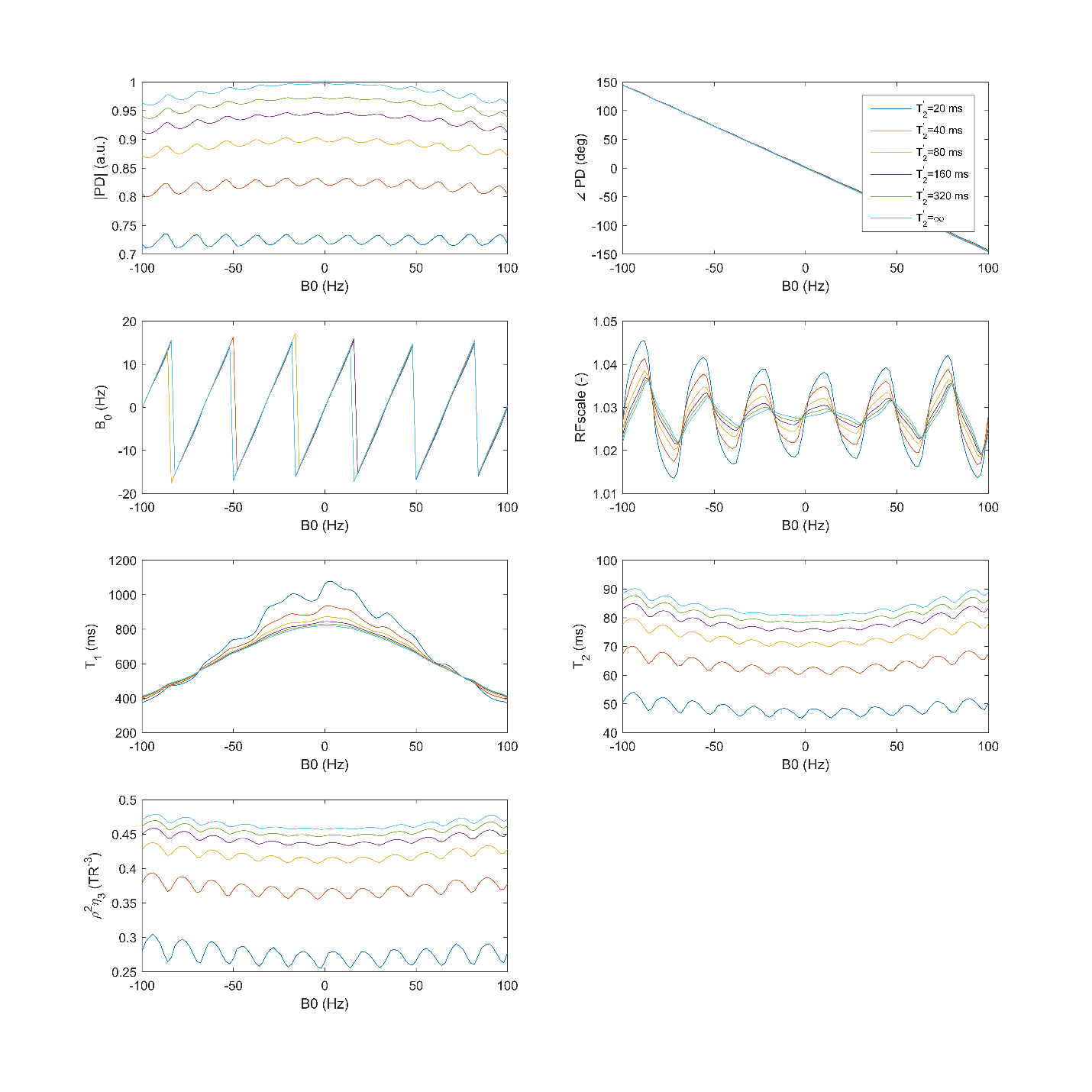


Figure S2: Parameters estimated from the data simulated including slice profile and for different $T_{2}^{'}$ as function of B_0_.
